# Surface‐Mediated Spin Locking and Thermal Unlocking in a 2D Molecular Array

**DOI:** 10.1002/advs.202300223

**Published:** 2023-05-18

**Authors:** Iulia Cojocariu, Andreas Windischbacher, Daniel Baranowski, Matteo Jugovac, Rodrigo Cezar de Campos Ferreira, Jiří Doležal, Martin Švec, Jorge Manuel Zamalloa‐Serrano, Massimo Tormen, Luca Schio, Luca Floreano, Jan Dreiser, Peter Puschnig, Vitaliy Feyer, Claus M. Schneider

**Affiliations:** ^1^ Peter Grünberg Institute (PGI‐6) Forschungszentrum Jülich GmbH 52428 Jülich Germany; ^2^ Elettra‐Sincrotrone S.C.p.A, S.S. 14 – km 163.5 Trieste 34149 Italy; ^3^ Dipartimento di Fisica Università degli Studi di Trieste via A. Valerio 2 Trieste 34127 Italy; ^4^ Institute of Physics University of Graz NAWI Graz, Universitätsplatz 5 Graz 8010 Austria; ^5^ Institute of Physics Czech Academy of Sciences Cukrovarnická 10/112 Praha 6 CZ 16200 Czech Republic; ^6^ ESISNA Group Instituto de Ciencia de Materiales de Madrid (ICMM‐CSIC) Sor Juana Inés de la Cruz 3 Madrid 28049 Spain; ^7^ CNR‐IOM Lab. TASC S.S. 14km 163,5 Trieste 34149 Italy; ^8^ Swiss Light Source Paul Scherrer Institut CH‐5232 Villigen PSI Switzerland; ^9^ Faculty of Physics and Center for Nanointegration Duisburg‐Essen (CENIDE) University of Duisburg‐Essen D‐47048 Duisburg Germany; ^10^ Department of Physics and Astronomy UC Davis Davis CA 95616 USA

**Keywords:** metalorganic layers, spin switch, stability, storage, surface

## Abstract

Molecule‐based functional devices may take advantage of surface‐mediated spin state bistability. Whereas different spin states in conventional spin crossover complexes are only accessible at temperatures well below room temperature, and the lifetimes of the high‐spin state are relatively short, a different behavior exhibited by prototypical nickel phthalocyanine is shown here. Direct interaction of the organometallic complex with a copper metal electrode mediates the coexistence of a high spin and a low spin state within the 2D molecular array. The spin state bistability is extremely non‐volatile, since no external stimuli are required to preserve it. It originates from the surface‐induced axial displacement of the functional nickel cores, which generates two stable local minima. Spin state unlocking and the full conversion to the low spin state are only possible by a high temperature stimulus. This spin state transition is accompanied by distinct changes in the molecular electronic structure that might facilitate the state readout at room temperature, as evidenced by valence spectroscopy. The non‐volatility of the high spin state up to elevated temperatures and the controllable spin bistability render the system extremely intriguing for applications in molecule‐based information storage devices.

## Graphical abstract

The working mechanism of molecular spintronic devices is based on an intrinsic property of electrons, thereby offering fast operations, low power consumption, and long retention time. While commonly the spin bistability in molecular complexes is only accessible at low temperatures with relatively short lifetimes, the system here presented reveals a distinct behavior.

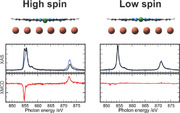

## 1 Introduction

With striking examples ranging from quantum teleportation^[^ ^1^ ^]^ to atomic‐scale nuclear spin imaging,^[^ ^2^ ^]^ tunable solid state spins^[^ ^3^, ^4^, ^5^ ^]^ form an important platform for information science. Because it provides straightforward single‐spin readouts, the optical spin interface of these solid‐state systems is crucial for a wide range of applications, from nanoscale sensing to long‐distance quantum communication. However, synthetic tunability of optical and spin properties, as well as the deterministic design of multibit arrays, remain challenging goals for this family of bits.

Chemical synthesis of molecular spin systems, on the other hand, allows for bottom‐up bit design.^[^ ^6^, ^7^ ^]^ Since the bit is not constrained to a specific host, a chemical approach provides tunability via atomistic control over the bit, scalability through a chemical assembly of extended structures, and portability across diverse environments (e.g., solution, surface, and solid‐state). These characteristics give effective control over the intrinsic and extrinsic surroundings of the molecular bits. Notably, nuclear spins may be controllably positioned within the molecular bit using chemical synthesis,^[^ ^8^ ^]^ and spin arrays can be produced in 1D‐, 2D‐, and 3D architectures,^[^ ^9^, ^10^ ^]^ and molecular spins can also be integrated into electronic and photonic devices.^[^ ^11^, ^12^ ^]^

In the field of on‐surface coordination chemistry, spin, and electronic state manipulation is possible through various approaches ranging from axial coordination through an internal^[^ ^13^ ^]^ or external ligand^[^ ^14^, ^15^, ^16^, ^17^, ^18^ ^]^ to doping^[^ ^19^ ^]^ and conformational changes.^[^ ^13^ ^]^ Often the changes between bistable electronic states at the interface in molecular systems are induced by the voltage pulse using a scanning tunneling microscope (STM) tip.^[^ ^20^, ^21^, ^22^ ^]^ The recent progresses in the design of molecular switches induced by STM tip on a metal substrate have been summarized in the reported review,^[^ ^23^ ^]^ with particular attention dedicated to the molecular configuration, switching mechanism, and the role of van der Waals forces between the molecules and the metallic electrodes.

Concerning the macrocyclic compounds with a chelated metal ion, it is well known in the case of transition metal phthalocyanines (TMPcs) that, if the ionic radius of the ion is large enough, for example, Sn(II), the central metal is not stable in the plane of the molecule and SnPc can adapt two non‐equivalent conformations on the surface with the ion pointing toward and away from the substrate.^[^ ^24^ ^]^ Interestingly, both configurations coexist in the first and second layer of SnPc, while the upper layers are characterized by single configurations.^[^ ^25^ ^]^ In this context, the possibility of inducing a molecular switch has been demonstrated with the aid of various external stimuli, such as light,^[^ ^26^, ^27^ ^]^ temperature,^[^ ^28^ ^]^ and electric fields.^[^ ^14^, ^29^, ^30^ ^]^

However, tip‐induced state switching is not a trivial experiment especially when conducted on metallic surfaces since probing itself can induce side effects such as cross‐bending, as observed for SnPc on Ag(111).^[^ ^31^ ^]^

Nevertheless, axial displacement is hardly observed in TMPcs with a smaller radius of the chelated metal ion, for example, NiPc. These molecules commonly have only one stable conformational structure, that is, the planar form, where the TM ion is located within the macrocycle plane^[^ ^24^, ^32^ ^]^ with a single spin configuration that can be stabilized. By exploiting the well‐established strong interaction between metalorganic layers and Cu(100),^[^ ^15^, ^16^, ^17^ ^]^ we demonstrate the surface‐mediated coexistence of two stable spin state in the 2D molecular array, high spin, and low spin, with the former originating from the axial displacement of the central TM of NiPc. Our conclusions are mainly based on photoemission spectroscopy experiments that are known to be less perturbative methods as compared to the pulse switch via STM tip mentioned above. The observed spin state bistability appears to be extremely stable as annealing to elevated temperatures (575 K) is required to displace the central Ni ion and consecutively alter its spin state. The high spin state, which up to now has been reported for NiPc only in solution chemistry,^[^ ^33^ ^]^ is eminently non‐volatile up to elevated temperatures, so that the switch from the bistable to a full low spin configuration can be controllably induced by providing a high energy thermal stimulus. This change of the spin state leads to appreciable changes in the valence electronic structure, detectable even at room temperature.

## 2 Results and Discussion

A detailed comprehension of the changes induced by the contact of the NiPc molecule with a metal electrode may take advantage of a brief discussion of the freestanding, that is, the gas‐phase molecule. Structurally, NiPc is composed of four isoindole ligands that bind to the nickel ion via their pyrrolic nitrogen atoms. The *D_4h_* square planar coordination of Ni(II) in the freestanding NiPc lifts the five‐fold degeneracy of the Ni 3d atomic orbitals (AOs) resulting in a (*d*_xy_)^2^, (*d*_(xz,yz)_)^4^, (*d*_z2_)^2^, and (*d*_x2−y2_)^0^ configuration, which corresponds to a non‐magnetic d^8^ Ni(II) low spin state. The highest occupied and lowest unoccupied molecular orbitals (HOMO and LUMO) are mainly delocalized on the Pc macrocycle and are of a_1u_ and 2e_g_ character, respectively (see Figure S1, Supporting Information).

These gas‐phase properties of the NiPc molecules can indeed be observed spectroscopically at the interface by electronically decoupling the molecule from the substrate. This can be achieved, for instance, by using the (√2×2√2)R45 oxygen‐passivated Cu(100) surface as a supporting template, where the covalent nature of the Cu—O interaction yields a strong localization of the surface electrons inhibiting the charge transfer from the metal to the organic overlayer.^[^ ^34^, ^35^ ^]^ The Ni(II)Pc low spin configuration expected for the freestanding molecule is confirmed to be the most stable one on this decoupling template (see Figure S2, Supporting Information).

When brought into contact with a surface, the gas‐phase molecular properties can undergo strong modifications. As a general rule, the greater the interaction between the molecular overlayer and the substrate, the greater the magnitude of the expected changes, for example, unconventional spin states. For this reason, owing to its high reactivity and ability to hybridize with 2D metalorganic self‐assemblies, metallic copper is a good candidate as an electrode to test the possibility of inducing a spin switch in NiPc.

**Figure 1:**
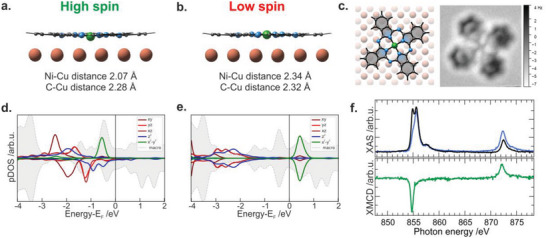
Simulated adsorption a,b) and electronic d,e) configuration of spin up and down Ni 3d states for the high spin and low spin conformation of NiPc interfaced to the Cu(100) substrate; the here reported pDOS calculations are based on the (7, ‐1; 3, 6) unit cell; c) Top view of the adsorption configuration along with the high‐resolution AFM image of a single NiPc molecule on Cu(100); f) XMCD spectra of monolayer NiPc/Cu(100) acquired at 3 K, while applying an external magnetic field of 6.8 T.

From a theoretical point of view based on density functional theory (DFT) calculations, this hypothesis proves to be valid: upon on‐surface deposition, the low spin (LS) state characteristic for the unsupported molecule switches to the high spin (HS) one. The HS configuration is stabilized by the out‐of‐plane displacement of the Ni ion (see **Figure** **1**a,b), and favored by 0.08 eV over the low spin system in the single molecule regime.

We further verify the geometry and adsorption site of the NiPc system on Cu(100) since the local adsorption geometry is known to strongly influence the molecule‐surface interaction and, consequently, could induce a different charge transfer mechanism and spin configuration of the chelated metal ion.^[^ ^36^ ^]^ To experimentally retrieve the adsorption site of NiPc, atomic force microscopy (AFM) measurements have been performed at low molecular coverage and the image is reported in Figure 1c together with the DFT‐based optimized adsorption geometry of NiPc on Cu(100) (see also Section S3, Supporting Information). The N—Ni—N axes in a single isolated NiPc molecule are rotated by ≈± 17 (2)° with respect to the [110] direction of the Cu(100) substrate, similar to the value reported for other MPcs.^[^ ^37^, ^38^, ^39^, ^40^, ^41^, ^42^ ^]^ As a consequence of the four‐fold symmetry of both Cu(100) substrate and NiPc, only these two azimuthally mirrored adsorption geometries are observed. The AFM data suggest that the hollow adsorption site on Cu(100) is favored, as for CuPc and ZnPc on Cu(100),^[^ ^41^, ^42^ ^]^ as it allows the NiPc molecule to move closer to the copper surface maximizing the molecular‐substrate interaction.^[^ ^17^ ^]^ The higher stability of this adsorption configuration compared to the top and bridge configuration is also confirmed by DFT calculations (see Section S3, Supporting Information for details).

The projected density of states (pDOS) versus *E*−*E*_F_ graph shows that for the Ni‐displaced Pc, the spin up and down Ni 3d states are significantly split (see Figure 1d,e). This leads to a sizable total magnetic moment, computed to be 1.3 µ_B_ for the d^9^ high spin system, independently of the unit cell considered in the calculation (see Figure S4, Supporting Information for the full range pDOS).

Experimentally, an evaluation of the magnetic properties of the nickel centers of Cu(100)‐supported NiPc in saturated coverage (1 ML) can be retrieved from the Ni L_3,2_‐edge X‐ray magnetic circular dichroism (XMCD) measurements. The absorption spectra acquired in normal incidence geometry with left and right circularly polarized light across the L_3_‐ and L_2_‐edge for the as‐deposited NiPc layer on Cu(100) are depicted in Figure 1f, together with the corresponding dichroic signal (green curve). The spectra of the as‐deposited NiPc, in particular the resonance at lower photon energy, show strong dichroism at the L_3,2_‐edge, supporting the surface‐mediated stabilization of a high spin state. The XMCD spectra have been collected in normal and grazing incidence, as well as at magic angle geometry (see Figure S5, Supporting Information). From the sum rule analysis performed using the evaluation approach from Ref.,^[^ ^43^, ^44^ ^]^ the orbital moment can be considered isotropic for the three considered geometries (m_L_ = 0.12 ± 0.03 µ_B_/n_h_), while the *m*_s,eff_ has a strong out‐of‐plane anisotropy with *m*_s,eff_ = 0.55 ± 0.05 µ_B_/n_h_ for *θ* = 0° and m_s,eff_ = 0.23 ± 0.03 µ_B_/n_h_ for *θ* = 70° (m_s,eff_ = 0.39 ± 0.04 µ_B_/n_h_ at magic angle). It is important to keep in mind the fact that the real magnetic moments can be only probed at full saturation, which may not be the case here (see magnetization curves acquired in the [−6.8; 6.8] T range, as reported in Figure S6, Supporting Information), and thus the expectation values of the moments represent a lower limit for the real magnetic moments in such scenarios. The impossibility of reaching saturation impedes the determination of the dipole term by angle‐dependent measurements, meaning that it is not possible to unequivocally determine the spin state based on the experimental data. Even if the real *m*_s,eff_ at full saturation could be slightly larger, and the dipole term could be negative,^[^ ^45^ ^]^ the experimentally retrieved value is about half of the calculated magnetic moment of 1.3 µ_B_ for the d^9^ high spin system. The coexistence of HS and LS species in similar amounts within the saturated molecular layer of NiPc on Cu(100) would explain this discrepancy between the calculated and measured values.

**Figure 2:**
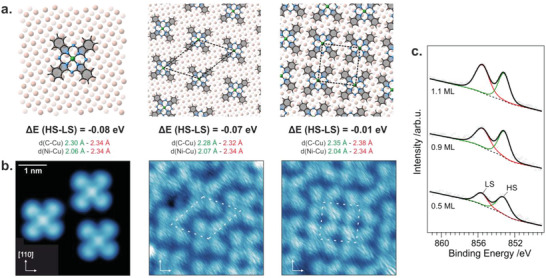
a) Coverage‐dependent DFT calculations for the single‐molecule and increasing molecular packing densities. The energy difference between the HS and LS states together with the optimized adsorption distances are given below each model; b) Corresponding STM images of the configurations considered above (the scanning parameters are reported in the Supporting Information); c) Coverage‐dependent XPS spectra acquired at the Ni 2p_3/2_ core level along with the fit of the HS and LS components.

To evaluate this observation and test our hypothesis, we model different molecular packing densities observed in the STM image of a close‐to‐saturation molecular coverage (see **Figure** **2**a), focusing on the effect of intermolecular interactions on the energy difference between the HS and LS states going from the single molecule regime toward a saturated highly packed 2D molecular layer. The large area STM image measured for almost saturated molecular coverage is reported in Figure S7 (Supporting Information), while the images for selected regions corresponding to the most common molecular arrangements are reported in Figure 2b, together with the molecular unit cells used in the calculation. The present theoretical investigation shows that, while the HS configuration is favored over the LS system in the single molecule regime by 0.08 eV, upon increasing the molecular packing density, the calculated energy difference between the HS and LS states reduces first to 0.07 eV, and then to only 0.01 eV when the molecules are adsorbed in a closely packed arrangement corresponding to saturated coverage.

To confirm this theoretically suggested trend of coexistence of NiPc species in HS and LS configuration in the dense layer we perform coverage‐dependent x‐ray photoemission spectroscopy (XPS) measurements of the Ni 2*p*_3/2_ core level. XPS is a suitable technique to obtain the chemical composition of the studied species at the interface based on the semi‐quantitative analysis of the peak area of photoemission lines observed in the spectra.^[^ ^46^ ^]^ The Ni 2*p*_3/2_ spectra reported in Figure 2c show two distinct photoemission lines with the maximum intensity at 852.8 ± 0.2 and 853.14 ± 0.2 eV. Following the interpretation of the Ni 2*p*_3/2_ core level measured for Ni tetraphenyl porphyrin adsorbed on bare and oxygen‐modified Cu(100),^[^ ^47^ ^]^ we assign the two peaks observed in the coverage‐dependent spectra of NiPc on Cu(100) as follows: the lower binding energy feature is associated with HS with d^9^ configuration while the second peak, at higher binding energy, is assigned, instead, to Ni(II)Pc with low spin d^8^ configuration. Semi‐quantitative analysis, that is, the evaluation of the areas of the two photoemission lines, is consistent with the theoretical trend mentioned above, namely that species with the HS configuration are more abundant compared to the LS configuration in the less dense layer, although in saturated coverage almost equal numbers of molecules with both spin configurations are observed.

Both the DFT calculation and XPS experiment fully support our initial hypothesis based on the XMCD analysis about the coexistence of two stable spin states for the chelated metal ion in the 2D NiPc array on the Cu(100) electrode.

**Figure 3:**
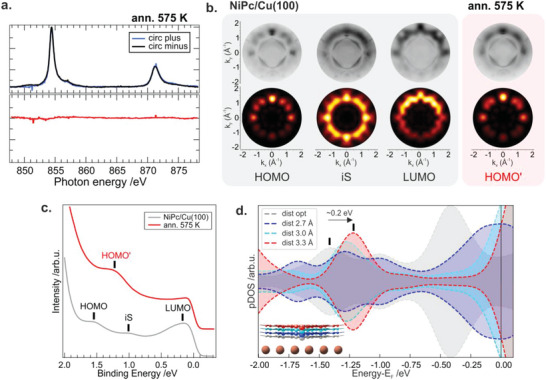
a) XMCD spectra of NiPc/Cu(100) acquired at 3 K, while applying an external magnetic field of 6.8 T after annealing to 575 K; b,c) Angle‐integrated valence band photoemission spectra and corresponding theoretical and experimental momentum maps acquired for the NiPc/Cu(100) before and after annealing to 575 K; d) pDOS calculation for different molecular‐substrate distances.

Next, we investigated the thermal robustness of the HS configuration observed in the pristine NiPc layer. The result shows that the high spin configuration is stable up to relatively elevated temperatures due to the surface pinning, resulting in an extremely non‐volatile state. Only upon annealing to 575 K, the HS state can be switched. The low spin Ni(II) state is stabilized by the axial displacement of the nickel ion, which, in this configuration, is coplanar with the macrocycle (see Figure 1b). This leads to a decrease of the Ni—N bond length, from 2.01 to 1.94 Å upon HS to LS switching. The reduced bond length implies an increased ligand field which, ultimately, leads to the stabilization of the low spin configuration. The simulated pDOS of the low spin state evidence the degeneracy of the spin up and down Ni 3d states (Figure 1e). This results in a vanishing magnetic moment, as confirmed by the absence of an XMCD signature for the system stabilized after annealing in all the experimental geometries probed (see **Figure** **3**a).

To obtain further insight into the energy level alignment of the occupied frontier MOs of the two forms, that is, pristine and annealed interfaces, Photoemission Orbital Tomography (POT) experiments have been performed. While STM and AFM provide information about the local density of states (DOS) and topography (including the adsorption site) of the molecular species, POT gives information about the energy level alignment averaged over a larger scale within the molecular assembly and azimuthal orientation of the molecules with respect the high symmetry direction of the substrate. In particular, the reciprocal space mapping of the MOs with POT allows us to directly identify the frontier MOs. Information regarding charge transfer phenomena occurring at the organic‐metal interface provides feedback on the theoretical calculations. The angle‐integrated valence band spectra acquired for as‐deposited NiPc on Cu(100) show the presence of three peaks, located at binding energies (BEs) of 0.2 ± 0.05, 1.0 ± 0.05, and 1.6 ± 0.05eV. To identify the origin of the features observed in the spectra the experimental momentum maps are compared to the square modulus of the Fourier transform (FT) of the real space molecular orbitals, calculated for the NiPc molecule. Within this approach, a one‐to‐one correspondence between the momentum distribution of the photocurrent and the MOs in the reciprocal space can be established.^[^ ^48^, ^49^, ^50^ ^]^ Consequently, the features observed at 0.2 and 1.6 eV for the pristine NiPc/Cu(100) interface system can be assigned to the former lowest unoccupied molecular orbital (LUMO) and highest occupied molecular orbital (HOMO) of the NiPc molecule, respectively, while the intermediate state (iS) at 1.0 eV of BE arises from the superposition of the HOMO and LUMO levels and is enhanced by the surface‐induced hybridization of the LUMO with the copper substrate (see Figure 1). Based on the present simulation of molecular maps the NiPc molecules are mirrored by 15 ± 5° with respect to the [110] high symmetry direction of the Cu(100) substrate, in good agreement with the AFM data discussed above. Upon annealing of the NiPc/Cu(100) interface to 575 K, both molecular features associated with HOMO and LUMO in the valence band spectra are shifted by 0.2 eV toward the metal Fermi level, while no significant variations on the azimuthal angle are observed. To get insight into the observed energy shift in the valence band spectra further DFT calculations have been performed. While the energy alignment of the frontier orbital is essentially independent of the position of the chelated metal ion with respect to the copper substrate in the configurations reported in Figure 1 (see Figure 1, Ni‐down and Ni‐in‐plane configuration), the position of the HOMO and LUMO features is strongly influenced by the molecule‐substrate distance. Indeed, our DFT calculations indicate that already an upward shift of the macrocycle backbone by ≥0.4 Å (depending on the unit cell used in the calculation, see Section S8, Supporting Information) leads to a downshift of −0.2 eV of the HOMO and LUMO levels, in good agreement with the experimental observations. These changes in the height lead also to a decrease in the DOS of the iS feature associated with the molecule‐surface hybridization at the interface, as observed in the valence spectrum acquired after annealing. Based on the computational and experimental evidence provided by the valence band spectra we suggest that after annealing of the NiPc/Cu(100) interface the distance between the substrate and the macrocycle plane is increased, while the azimuthal orientation of the molecular species remains unchanged.

The decreasing of the molecule‐substrate interaction after annealing, going to a more decoupled molecular system is also elaborated in the analysis of the NEXAFS spectra measured across the N K‐edge (see Section S9, Supporting Information). Moreover, the STM measurements show that after annealing to the temperature above 575 K molecules spontaneously form new supramolecular structures (see Figures S10,S11, Supporting Information) similar to ZnPc on the Cu(100) surface.^[^ ^42^ ^]^ As the distance between the adjacent molecules in newly formed supramolecular structures is smaller than in the densely packed molecular layer reported in Figure 2, we suggest the formation of chemical bonds between the connected NiPc molecules within the observed structures rather than Van der Waals or hydrogen bond interaction. Moreover, similar high temperatures (close to 575 K) have been reported for on‐surface reactions in the metal porphyrin molecules on the metal substrate.^[^ ^45^, ^51^ ^]^ The formation of the chemical bonds in the formed new molecular structures after annealing to 575 K would explain the irreversibility of the spin transition in Ni chelated ion of phthalocyanine after cooling down the interface back to the pristine temperature.

## 3 Conclusion

Commonly, the coupling between the transition metal Pc and the metal substrate stabilizes the chelated ion in a single spin state configuration, because of the interaction occurring between the 3d shell and the substrate atoms. In contrast, here we show that the molecular structural properties, that is, the position of the transition metal ion with respect to the macrocycle plane, are strongly correlated with the magnetic states, which generate two stable local minima with high and low spin configurations. By using a combination of DFT‐based calculations and experimental methods, we revealed that the axial displacement of the Ni ion from the pristine gas‐phase in‐plane coordination can be induced within the on‐surface coordination chemistry approach by exploiting the relatively high reactivity of the copper electrode, thereby locking the nickel ion in spin state bistability. The magnetic HS state and LS state coexist in the pristine layer and can be switched to the non‐magnetic LS state by a thermal stimulus at the interface. These changes in molecular magnetism are correlated with changes in the electronic structure that could facilitate the reading out of the spin state even at room temperature.

The binary magnetic configuration of the NiPc/Cu(100) interface provides a case study for its application in molecule‐based devices designed for information storage. Indeed, the two non‐volatile and controllable states in the pristine NiPc that are accessible as a consequence of the given contact between the molecular layer and the copper electrode can be visualized as the 0 and 1 positions of the molecular bit required for data storage devices.

## 4 Experimental Section

### Sample Preparation

The Cu(100) surface was prepared by several cycles of Ar^+^ sputtering at 2.0 keV and subsequent annealing of the sample up to 800 K. NiPc molecules (Sigma–Aldrich, purity 95%) were thermally sublimated at 650 K from a Knudsen cell type evaporator onto the copper substrate kept at room temperature. At the ALOISA beamline, the evaporation rate was checked with a quartz microbalance. The oxygen‐reconstructed surface was prepared by dosing 800 L of O_2_ while keeping the substrate constantly at 500 K,^[^ ^52^ ^]^ and the (√2 × 2√2)R45° reconstruction was checked by LEED in all the experimental setups.

### X‐Ray Magnetic Circular Dichroism

The difference between two absorption spectra measured with circularly polarized X‐ray light with opposite helicities (µ_+_ and µ_‐_) was evaluated at the L_3_ and L_2_ absorption edges in order to check for a spin‐polarization of the empty states. The measurements were carried out at the EPFL/PSI X‐Treme beamline, at the Swiss Light Source, Paul Scherrer Institut, Villigen, Switzerland.^[^ ^53^ ^]^ They were performed in the total electron yield mode, measuring the drain current of the sample. A magnetic field of 6.8 T was applied parallel to the light propagation in order to maximize the signal, and the sample was rotated in order to measure the absorption with normal (*θ* = 0°) and grazing (*θ* = 70°) incidence. The sample temperature for all the spectra presented here was ≈3 K.

### NEXAFS and XPS

The near‐edge X‐ray absorption fine structure and X‐ray photoemission spectra were measured at the ALOISA beamline^[^ ^54^ ^]^ of the Elettra Synchrotron in Trieste, Italy. At ALOISA, the sample was mounted at grazing incidence on a manipulator coaxial to the photon beam. The XPS spectra were measured at a grazing angle of 4˚ in transverse magnetic polarization (quasi p‐pol) with the electron spectrometer in normal emission. The total electron energy resolution (analyzer and beamline) was set to 300 meV. The NEXAFS spectra were measured in the partial electron yield mode using a channeltron multiplier equipped with a front grid polarized at a negative bias to reject low‐energy secondary electrons (−820 V for the Ni L_2_‐edge and −370 V for the N K‐edge). The polarization was changed from p‐pol to transverse electric (s‐pol) by rotating the sample around the photon beam axis while keeping the sample at a constant grazing angle (and illuminated area) of 6˚.

### Photoemission Orbital Tomography

The POT experiments were performed at the NanoESCA beamline of Elettra, the Italian synchrotron radiation facility in Trieste, using an electrostatic photoemission electron microscope (PEEM) set‐up described in detail in Ref.^[^ ^55^ ^]^ The data were collected with a photon energy of 30 eV and a total energy resolution of 100 meV, using p‐linearly polarized light.

### STM and ncAFM

STM and ncAFM experiments were performed in an ultrahigh vacuum system (UHV) with a based pressure of 5 × 10^−11^ mbar. The SPM scanner and ncAFM sensor (Createc GmbH) were placed in a cryogenic bath at 6 K. The q‐plus sensor consists of a 30 kHz mechanical oscillator with a Pt—Ir tip, sharpened ex‐situ by a focused Xe ion beam (Tescan FERA3). The STM images were taken at constant current regime. The ncAFM frequency shift images were recorded with an oscillating amplitude of 50 pm. In a separate chamber, the sample was prepared with the same conditions as described above in the sample preparation section.

### Computational Methods

All calculations were performed within the framework of DFT+U,^[^ ^56^ ^]^ where the Ni d‐orbitals were corrected with an effective Hubbard U_eff_ of 3 eV. The structures were optimized for utilizing the Vienna Ab Initio Simulation Package (VASP)^[^ ^57^, ^58^ ^]^ version 5.4.4. Exchange‐correlation effects were approximated by the functional of Perdew–Burke–Ernzerhof (PBE)^[^ ^59^ ^]^ and Van der Waals contributions were treated with Grimme's D3 dispersion correction.^[^ ^60^ ^]^ The projector‐augmented wave (PAW) method was used,^[^ ^61^ ^]^ assuming an energy cutoff of 400 eV. As no predominant experimental unit cell was detected, Three molecular arrangements (Figure S8, Supporting Information) covering the range from a very tightly packed monolayer to an isolated molecule on the surface were modeled. Depending on the size of the unit cell, the structures were calculated on an adjusted Monkhorst–Pack^[^ ^62^ ^]^ grid of (6 × 6 × 1), (4 × 4 × 1), or (3 × 3 × 1) *k‐*points. The surface was simulated within the repeated slab approach using four metallic layers and a 30 Å vacuum layer. To prevent disturbing spurious electrical fields, a dipole layer was placed in the vacuum region.^[^ ^63^ ^]^ During the optimization, the two bottom Cu layers of the slab were constrained and the ionic positions calculated until the remaining forces were below 0.01 eV Å^−1^. Furthermore, the z‐coordinate of the molecule was constrained during the evaluation of the distance dependence.

For the simulation of the XPS spectra, we utilized the GPAW code (version 21.1.0).^[^ ^64^, ^65^ ^]^ The structures were recalculated at the same level of theory applying the delta Kohn–Sham total energy differences method (ΔKS). According to this scheme, the energies of the C 1s core level excitations are determined as the total energy differences between the ground state and the first core ionized states.^[^ ^66^ ^]^ For the ionized states, the core electrons of each target atom were modeled by a C 1s core‐hole setup, while a compensating homogeneous background was introduced to ensure the neutrality of the periodic unit cells. While the Kohn–Sham procedure should give consistent results for all atoms of the same kind, the absolute binding energies depend on the exchange‐correlation functional. Therefore, the calculated energy scale was rigidly shifted to align with the experiment.

## Conflict of Interest

The authors declare no conflict of interest.

## Supporting information

Supporting Information

## Data Availability

The data that support the findings of this study are available from the corresponding author upon reasonable request.
