# Supplementary material for: Surface‐Mediated Spin Locking and Thermal Unlocking in a 2D Molecular Array
Source: Adv Sci (Weinh). 2023 May 18;10(22):2300223. doi: 10.1002/advs.202300223 (PMC10401090; doi:10.1002/advs.202300223)
Supplement: Supplementary file 1 — Supporting Information [file ADVS-10-2300223-s001.pdf]

ADVANCED SCIENCE
Open Access

Supporting Information

for *Adv. Sci.*, DOI 10.1002/advs.202300223

Surface-Mediated Spin Locking and Thermal Unlocking in a 2D Molecular Array

Iulia Cojocariu, Andreas Windischbacher, Daniel Baranowski, Matteo Jugovac, Rodrigo Cezar de Campos Ferreira, Jiří Doležal, Martin Švec, Jorge Manuel Zamalloa-Serrano, Massimo Tormen, Luca Schio, Luca Floreano, Jan Dreiser, Peter Puschnig, Vitaliy Feyer* and Claus M. Schneider*

Supporting Information for "Surface-mediated spin locking and thermal unlocking in a two-dimensional molecular array"

Iulia Cojocariu,[1,2,*] Andreas Windischbacher,[3] Daniel Baranowski,[1] Matteo Jugovac,[1,2] Rodrigo Cezar de Campos Ferreira,[4] Jiří Doležal,[4] Martin Švec,[4] Jorge Manuel Zamalloa Serrano,[5] Massimo Tormen,[6] Luca Schio,[6] Luca Floreano,[6] Jan Dreiser,[7] Peter Puschnig,[3] Vitaliy Feyer,[1,8,*] Claus M. Schneider[1,8,9]

1. Peter Grünberg Institute (PGI-6), Forschungszentrum Jülich GmbH, 52428 Jülich, Germany
2. Elettra-Sincrotrone, S.C.p.A, S.S 14 - km 163.5, 34149 Trieste, Italy
3. Institute of Physics, University of Graz, NAWI Graz, Universitätsplatz 5, 8010 Graz, Austria
4. Institute of Physics, Czech Academy of Sciences, Cukrovarnická 10/112, Praha 6 CZ16200, Czech Republic
5. ESISNA Group, Instituto de Ciencia de Materiales de Madrid (ICMM-CSIC), Sor Juana Inés de la Cruz 3, 28049 Madrid, Spain
6. CNR-IOM, Lab. TASC, s.s. 14 km 163,5, 34149 Trieste, Italy
7. Swiss Light Source, Paul Scherrer Institüt, CH-5232 Villigen PSI, Switzerland
8. Faculty of Physics and Center for Nanointegration Duisburg-Essen (CENIDE), University of Duisburg-Essen, D-47048 Duisburg, Germany
9. Department of Physics and Astronomy, UC Davis, Davis, CA 95616, USA

Emails: i.cojocariu@fz-juelich.de, v.feyer@fz-juelich.de.

S1. Gas-phase DFT calculated pDOS

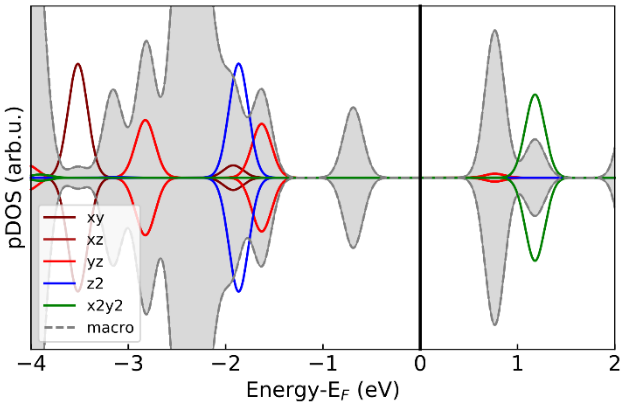

Figure S1. Computed pDOS for the gas-phase NiPc molecule.

S2. Ni L$_3$-edge NEXAFS

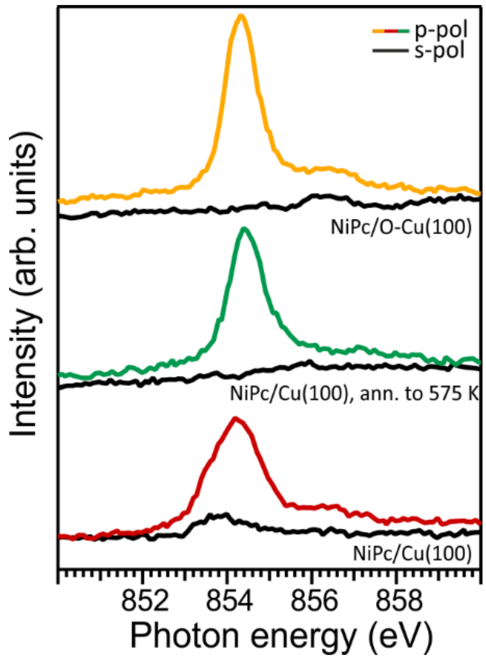

Figure S2. Ni L$_3$-edges NEXAFS acquired using both linearly p- and s-polarized light for NiPc/Cu(100) before and after annealing to 575 K and for the NiPc/O-Cu(100) systems.

S3. Adsorption site determination

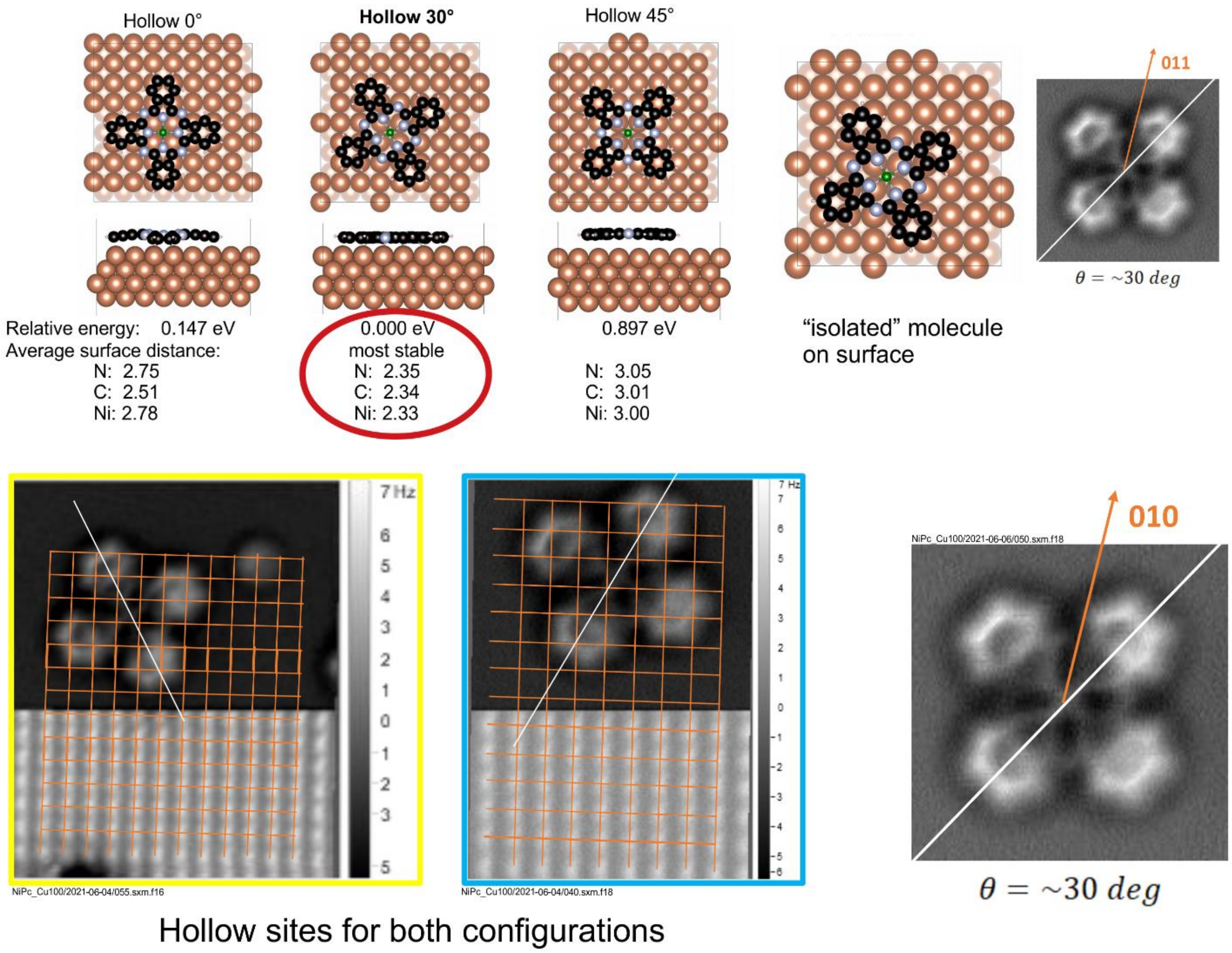

Figure S3. DFT-based optimization of the adsorption geometry at the single-molecule regime and corresponding AFM image.

DFT calculations on the adsoption site

For defining the most favored adsorption geometry of NiPc on the Cu(100) surface, we consider the three common adsorption sites, *i.e.* "top", "bridge" and "hollow". The adsorption site are in reference to the Ni center. If not stated otherwise, values in the table are for LS species. The molecule favors configurations where the macrocycle rings lie above a "hollow" site (top45, hollow0, hollow30). These configurations show the strongest interaction between the molecule and the surface (i.e., smallest molecule-surface distance). The Ni adsorbing on a hollow site and the molecule rotated by 30° to the main crystal direction [110] seems to be the most favored, as the molecule can lie almost perfectly flat on the surface. This finding is also supported by the angle analysis from ARPES data.

Table S1. Relative energies of NiPc adsorption sites with molecules rotated in various angles.

Adsorption site (rotation)	Relative energy /eV
Top (0)	0.87
Top (30)	1.01*
Top (45)	0.11
Bridge (0)	0.58
Bridge (30)	0.66*
Bridge (45)	0.56
Hollow (0)	0.15
Hollow (30)	0.00 (spin: -0.07)
Hollow (45)	0.90

*These values correspond to structures, which were relaxed on loose convergence settings (forces below 0.05 eV/A). Tightening the convergence criteria to 0.01 eV/A revealed that these adsorption configurations are not local minima, but lie on a flat plateau region of the potential energy surface. With further optimization, Top (30) transitioned into Top (45) and Bridge (30) relaxed to Hollow (30).

Additionally for the “hollow (30)”, the Ni is close enough to interact with the surface, resulting in a spin on the Ni (additional energy gain by 0.07 eV). This interaction pulls the Ni out of the molecule towards the surface (from 2.35 Å to 2.07 Å), while at the same time the molecular macrocycle stays at approximately the same height (~2.3 Å). Our calculations, thus, have found two local minima for the same adsorption site, which differ in the spin of the system (spin and no-spin) and in the distortion of the molecule (Ni out and inside of macrocycle). Compared to the next best adsorption site, the spin-polarized species in “hollow (30)” is in total favored by ~0.2 eV.

In comparison with the adsorption behaviour of other MPcs on Cu(100), one might observe that the azimuthal angle of absorption is independent of the central metal ion, and it is reported to be around ± 23° relative to the [110] direction of Cu(100) for either H_2Pc[1] or FePc,[1,2] CoPc,[2,3] CuPc[4,5] and ZnPc.[6] However, the behavior differs when it comes to the adsorption site, and a trend can be observed as the central metal 3d occupation number increases. FePc (d^6) and CoPc (d^7) adsorb with the central metal at the top position of Cu(100), while for NiPc (d^8), CuPc (d^9) and ZnPc (d^{10}), the central metal resides in the hollow site. This behavior can be attributed to the increased stabilization of the 3d-like orbitals going from FePc to ZnPc:[7] while for FePc and CoPc, the first reduction occurs on the metal (since the 3d-like orbitals are located near the Pc HOMO), in contrast, the phthalocyanine macrocycle is reduced upon adding an electron on the other MPcs. Considering this, the adsorption site is defined by the energy gain from maximizing charge transfer on metal (top site) or Pc ligand (hollow site). Top-site adsorption disfavors axial displacement of the central metal, so in the case of FePc and CoPc, it is very unlikely to observe a similar phenomenon to the one we reported for NiPc. As for the molecules that adsorb in the hollow position, it should be noted that NiPc differs in some properties from CuPc and ZnPc: the HOMO-LUMO gap of NiPc is calculated to be 1.4 eV, as for FePc and CoPc, while CuPc and ZnPc have a gap around 2 eV. This fact, coupled with the higher 3d stabilization for CuPc and ZnPc, most likely disfavors the reduction of the central metal in the first place and, consequently, inhibits the spin switching.

S4. Full range pDOS for the d^9 high spin state

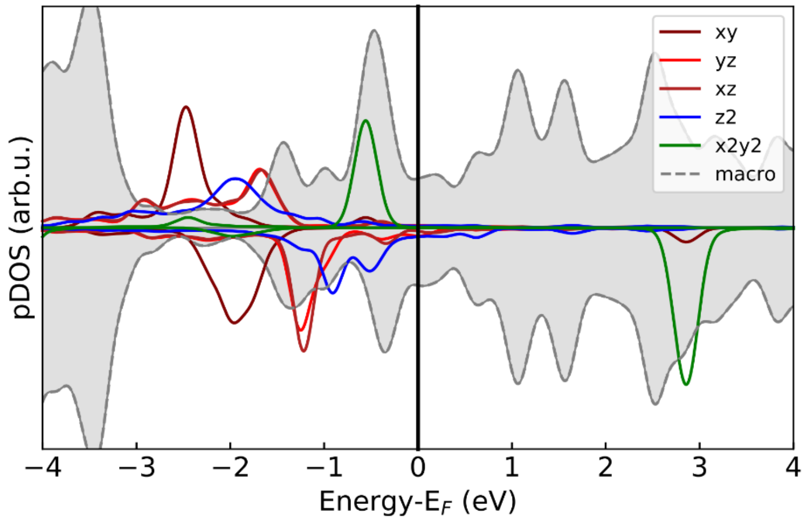

Figure S4. Full range computed pDOS for the d^9 high spin NiPc molecule.

S5. Angle-dependent XMCD

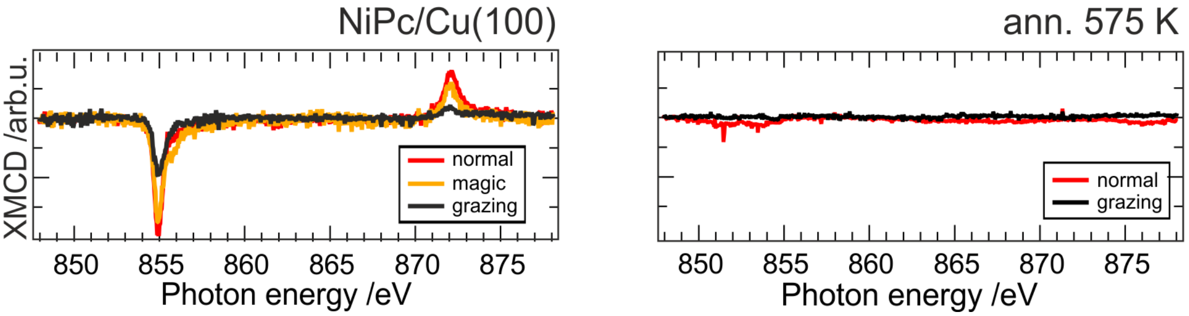

Figure S5. Angle-dependent XMCD spectra for NiPc on Cu(100) before and after annealing to 575 K. T=4 K, B=6.8 T.

S6. Magnetization curves of NiPc/Cu(100)

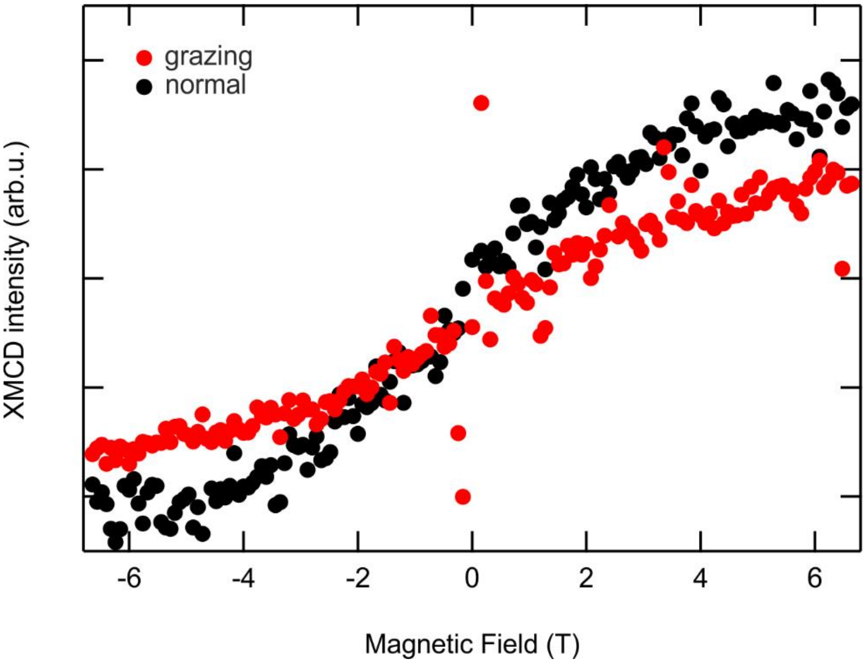

Figure S6. Magnetization curves acquired on the NiPc/Cu(100) system in grazing and normal incidence geometry. T=4 K.

S7. Large scale RT STM images on the saturated coverage

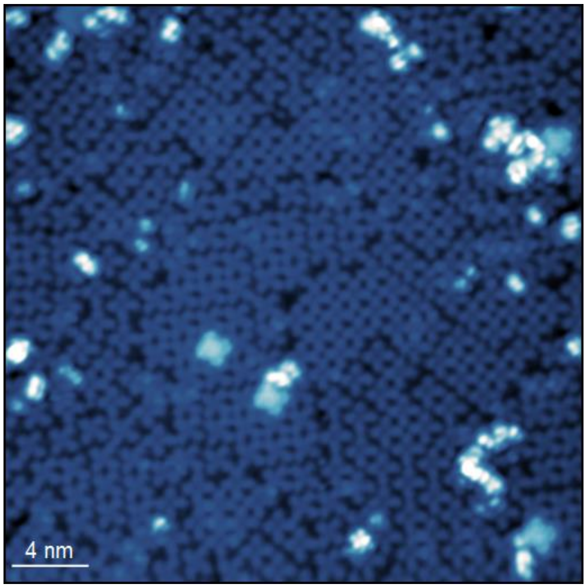

Figure S7. RT (300 K) STM images acquired for the as-deposited NiPc on Cu(100). Estimated coverage around 1 ML. U = 1 V, I = 20 pA, 30x30 nm^2

S8. Unit cell dependence

We modeled three unit cells of different size to test the dependence of our computational results on intermolecular interaction (Figure S3 top top, epitaxial matrices from left to right: $\begin{pmatrix}8 & -2\\ 2 & 8\end{pmatrix}$, $\begin{pmatrix}7 & -1\\ 3 & 6\end{pmatrix}$, $\begin{pmatrix}5 & -2\\ 2 & 5\end{pmatrix}$). Regardless of the coverage, the projected DOS shows an occupation of the degenerate LUMO of the molecule due to charge transfer from the surface. Another universal consequence of the interaction with the substrate is a broadening of the electron density between the former HOMO and LUMO states, in contrast to energetically localized states in a gas phase molecule. As shown in the main text, this overlap of HOMO and LUMO density then gives rise to the observed ARPES features. Furthermore, full geometry optimization favors a spin polarized solution in all three cases (Figure S3 middle, relative energy: E_{spin}-$E_{no\ spin}$), where the Ni atom gets displaced from the center of the macrocycle. Note, however, that the spin solution gets less favored with stronger intermolecular interactions in the tight packed arrangement, as it is more difficult for the molecules to distort and get close to the surface.

Moving NiPc away from the surface reduces the charge transfer and shifts its LUMO towards and above the Fermi level (Figure S3 bottom). Beyond an upward shift of 1Å, we then recover the energetically localized states as in the gas phase molecule (see Figure S1).

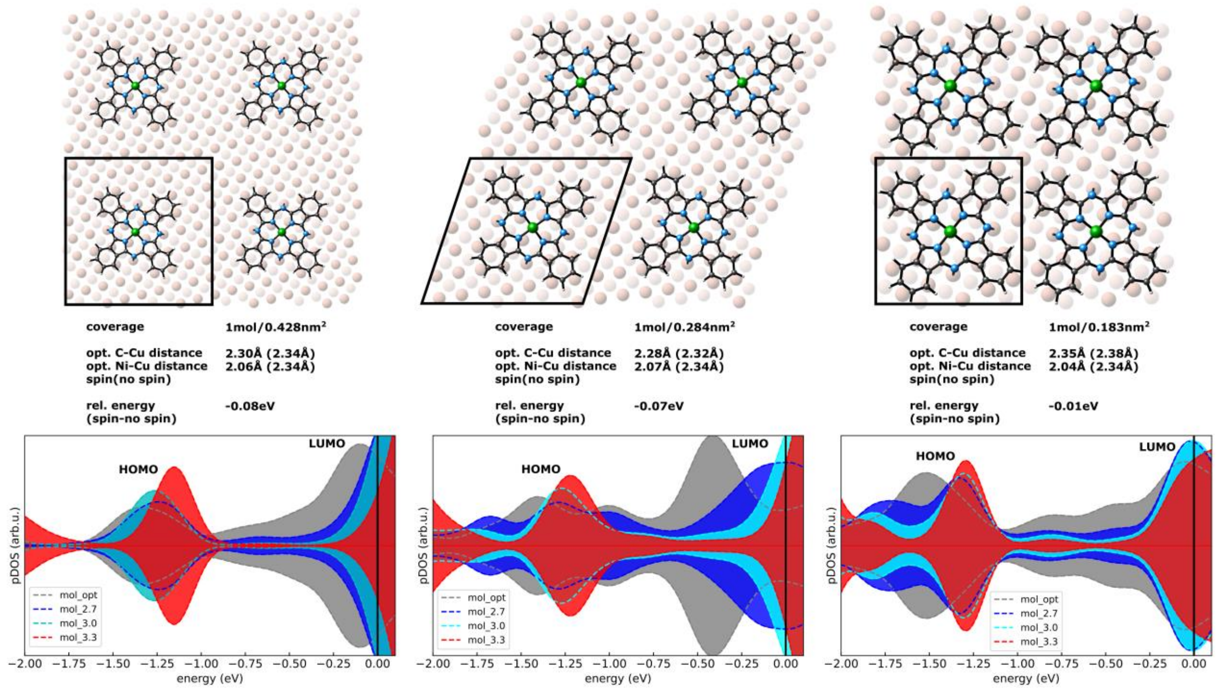

Figure S8. (top) Models of three different molecular arrangements ranging from an isolated molecule on the surface (left) to a very dense packing (right). (middle) Coverage, optimized adsorption heights and relative energy between the spin polarized and unpolarized solution of the three models. (bottom) Density of states projected onto the organic macrocycle (C, H, N atoms) as a sum over s-, p- and d-orbital projection. Additional to the optimized structure (grey), the molecule was shifted 0.4 (blue), 0.7 (cyan) and 1 Å (red) above the surface.

S9. N K-edge NEXAFS spectra

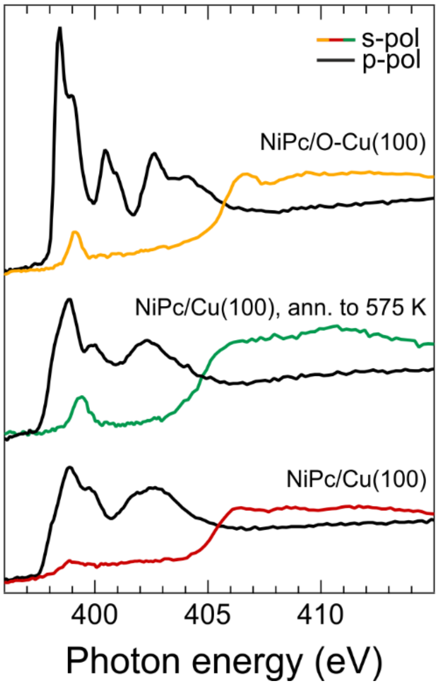

Figure S9. N K-edge NEXAFS spectra of NiPc monolayer on reference O-Cu(100) and Cu(100) before and after annealing to 575 K.

In Figure S9 are reported the N K-edge NEXAFS spectra of NiPc recorded before and after annealing to 575 K. In the spectra measured in p-polarization for the NiPc/Cu(100) system, the intense spectral features observed in the photon energy range of 397−404 eV can be assigned to the transition of N 1s electrons to the π*-symmetry unoccupied MOs, while the resonances above 404 eV are attributed to 1s →σ*-MO transitions. Moreover, the low-energy resonance in the spectra measured using s-polarization is assigned to the transition of N 1s electrons to the σ*-symmetry MO (sensitive to the N-ligand-to-Ni $3d_{x2-y2}$ bonding and backbonding).

The linear dichroism observed in the N K-edge spectra, *i.e.*, the maximum intensity of the π* transitions in p-polarization and the almost vanishing intensity of these resonances in the spectra measured in s-polarization, indicates that the molecule is highly oriented on the surface, with the molecular plane lying parallel to the substrate. As mentioned in the main text, the changes occurring in the electronic structure of the electrode-contacted molecules compared to gas phase-like species can be determined by comparing the spectra of adsorbed molecules with the reference data of electronically decoupled systems measured on the oxygen-passivated copper surface.[8] By comparison with the spectra of the NiPc/Cu-O interface, we observe that the decrease in intensity of the low energy π* and σ*-symmetry resonances in the spectrum of

pristine NiPc deposited on the Cu(100) supports the filling of the low energy π* MOs via the charge donation from the substrate to the adsorbed layer. Upon annealing to 575 K, the N K-edge spectra suggest a decrease of the charge transfer to the molecular macrocycle. These findings are supported also by the experimental and simulated C 1s core level spectra reported in section S15. The changes of the σ* transition, being sensitive to the metal-ligand bond, are further complementing this. The increase in intensity due to the decreased charge donation and the shift in energy support the axial displacement of the metal ion and the variation in the ligand strength indicated by the DFT simulations.

S10. RT STM before and after annealing of NiPc monolayer on Cu(100)

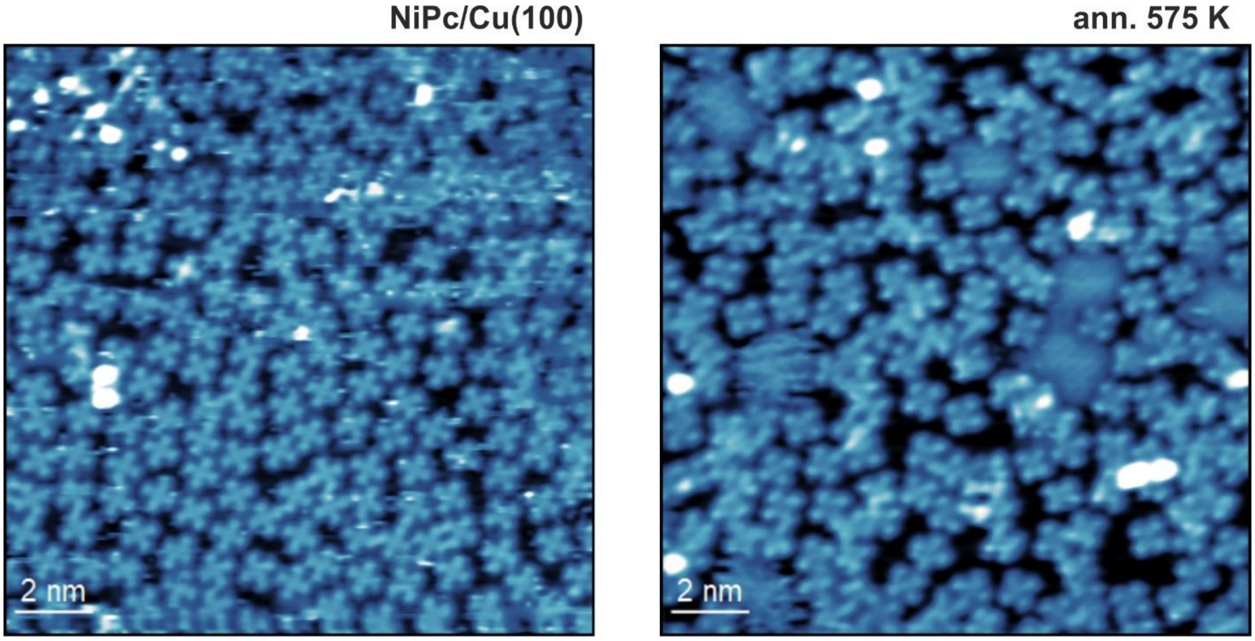

Figure S10. RT (300 K) STM images of NiPc on Cu(100) before (U=-635 mV, I=310 pA, 15x15 nm^2) and after annealing (U=-513 mV, I=160 pA, 15x15 nm^2) to 575 K. Estimated coverage around 0.9 ML.

S11. Zoom-in of LT-STM images and STS spectra at low coverage

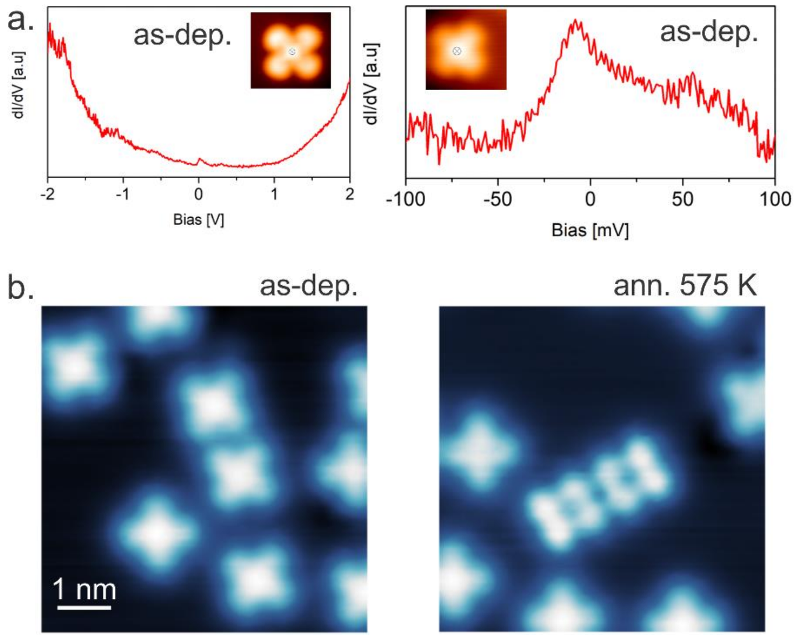

Figure S11. a) STS spectra acquired in the [-2,+2] V and [-0.1,+0.1] V ranges at the center of single NiPc molecules presented in the inset. U = 0.1 V, I = 50 pA, 2x2 nm^2; b) STM images (U = 0.1 V, I = 50 pA) acquired in the low coverage regime before and after annealing to 575 K. The occurrence of dimers showing a different appearance can be appreciated after annealing.

The dI/dV spectra recorded on the center of the single molecules (in correspondence with the chelated metal ion) show the presence of a feature centered around zero bias voltage, which *could* be associated with a Kondo feature as a result of electron transfer into the LUMO of NiPc, but to confirm it the magnetic response under a variable external magnetic field should be investigated and evaluated first, also at lower temperatures. It should be noted that this STS peak could, on the other hand, be ascribed to LUMO itself as the observed width is larger than those expected for a Kondo resonance[5]. While in the STM images acquired at zero bias voltage all the single molecules show a bright protrusion in the center, after annealing we observe the formation of sparse (in the low coverage regime) molecular dimers that manifest a completely different appearance, with a depression in the correspondence of the metal ion location. This change is probably associated with the switch to the LS state. This observation is further corroborated by the STM, XPS, XMCD and DFT results for the saturated NiPc coverage, where the coexistence of the HS and LS state is observed in the as-deposited layer, while upon annealing the maximization of the intermolecular interactions lead to the full conversion to an LS state.

S12. Calculated MOPDOS of high spin and low spin NiPc on Cu(100)

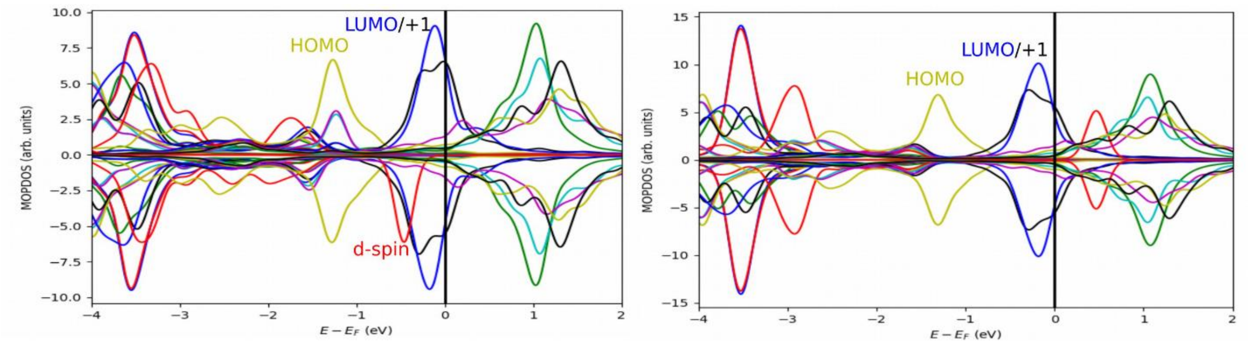

Figure S12. MOPDOS of the NiPc in the high-spin (left) and low-spin (right) configurations.

S13. Supporting LT STM images for the 0.3 ML NiPc on Cu(100)

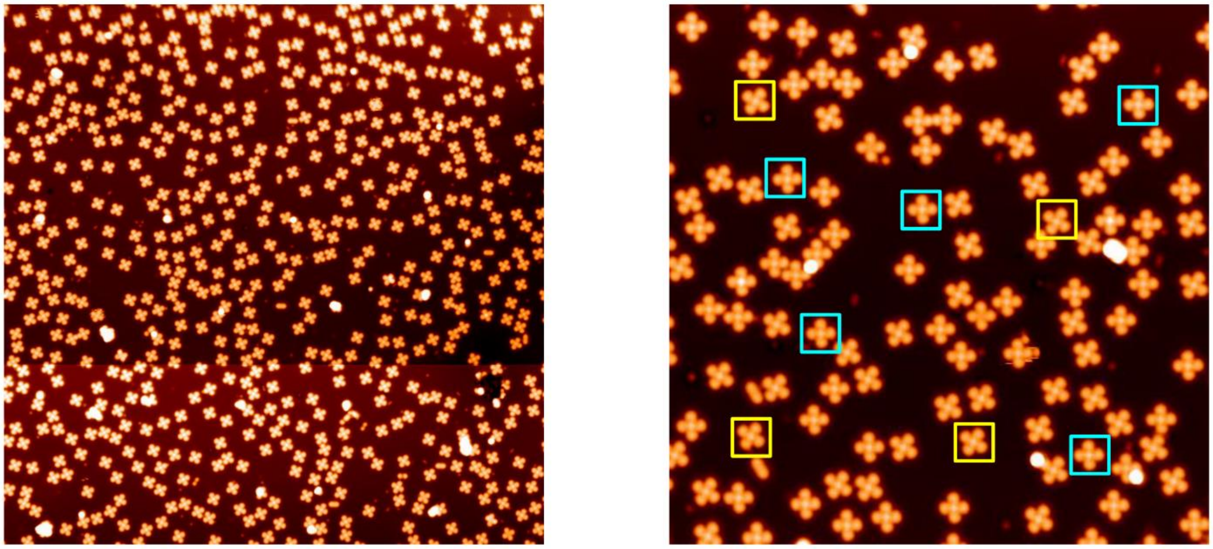

70x70 nm² (U=100mV , I=50pA) 30x30 nm² (U=100mV , I=50pA)

Figure S13a. LT (70 K) STM images acquired for the as-deposited NiPc on Cu(100). Estimated coverage around 0.3 ML. Blue and yellow boxes evidence the two azimuthally mirrored domains.

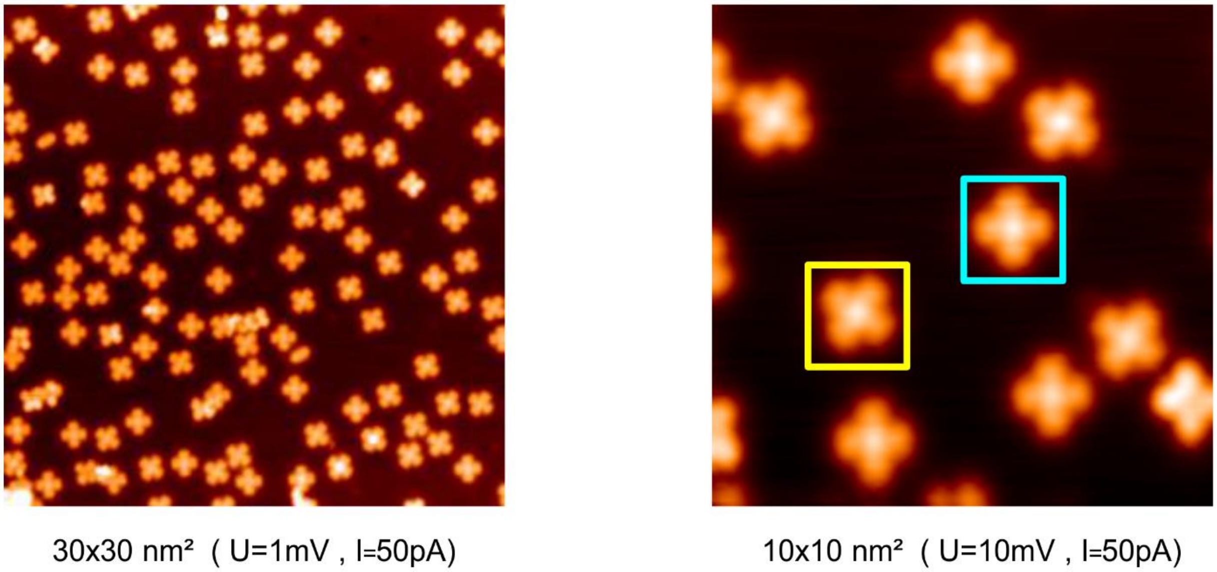

Figure S13b. LT (70 K) STM images acquired for the NiPc on Cu(100) system after annealing to 575 K. Estimated coverage around 0.3 ML. Blue and yellow boxes evidence the two azimuthally mirrored domains.

S14. C 1s XPS

Complementary to the NEXAFS and POT experiments, we have performed X-ray photoemission spectroscopy (XPS) to study the effect of annealing on the core levels of NiPc. As the frontier orbitals of NiPc, which are involved in the switchable substrate-molecule interaction, are mainly localized on the carbon macrocycle, we expect the largest influence on the C 1s spectra. To establish the main peaks in our NiPc C1s analysis, we start with the experimental data of the gas-phase-like NiPc on an oxygen-passivated Cu surface as a reference system (Figure S14a). The spectrum consists of two dominant peaks, which we attribute to emissions from carbons bound to other carbons (284.6 eV) and to nitrogen (286.0 eV), respectively. The small peak at 287.8 eV was previously identified as a $\pi\rightarrow\pi^*$ satellite.[9–11] In accordance with the stoichiometric ratio of C-C and C-N in NiPc (24:8), we obtain a ratio of the experimental peak intensities of 3:1. The electronic decoupling from the surface also allows us to compare the experimental spectra to C1s core-energies of the gas phase molecule calculated with the ΔK-S approach. Here, we find excellent agreement in the relative peak positions between experiment and theory, thus, supporting our assignment (Figure S14c top, the absolute calculated values are shifted for comparison).

Going from a passivated to a pure Cu surface, the as-deposited NiPc receives charge from the substrate, thereby shifting the degenerate LUMOs (LUMO and LUMO+1) below the Fermi

level. According to our calculations, the additional occupation of these orbitals increases especially the electron density at the C-N component (seen in Figure S14b). Assuming such a local charge transfer would also imply a shift of the respective C 1s component toward lower binding energies. Indeed, we see a decrease of the peak distance in the experimental XPS spectrum from 1.37 eV (O-Cu) to 1.10 eV (Cu) (Figure S14c middle). This relative shift between the two C components is also reproduced in the XPS simulation for the NiPc/Cu interface.

Upon annealing, the experimental XPS spectrum broadens and the two main peaks move further apart again (1.19 eV). We can explain these findings by the decrease of the electron density in the LUMO, pointing toward a reduced charge transfer between the NiPc and the surface. As also argued in the main text, we have simulated this case by artificially lifting the molecule upwards between 0.5 – 1 Å from its optimized adsorption height. Such geometries yield a similar relative shift in the simulated XPS spectrum as was found in experiment (Figure S14c bottom). Note that the Ni spin state switch at the initial equilibrium adsorption height has minor effects on the frontier orbitals (as can also be inferred from Figure 1 in the main text). Increasing the molecule-surface distance in the simulations is crucial to reduce the calculated charge transfer and, consequently, affect the C1s XPS spectrum. Thus, the comparison between XPS experiments and theory additionally supports our idea of a change in interaction between molecule and substrate upon annealing.

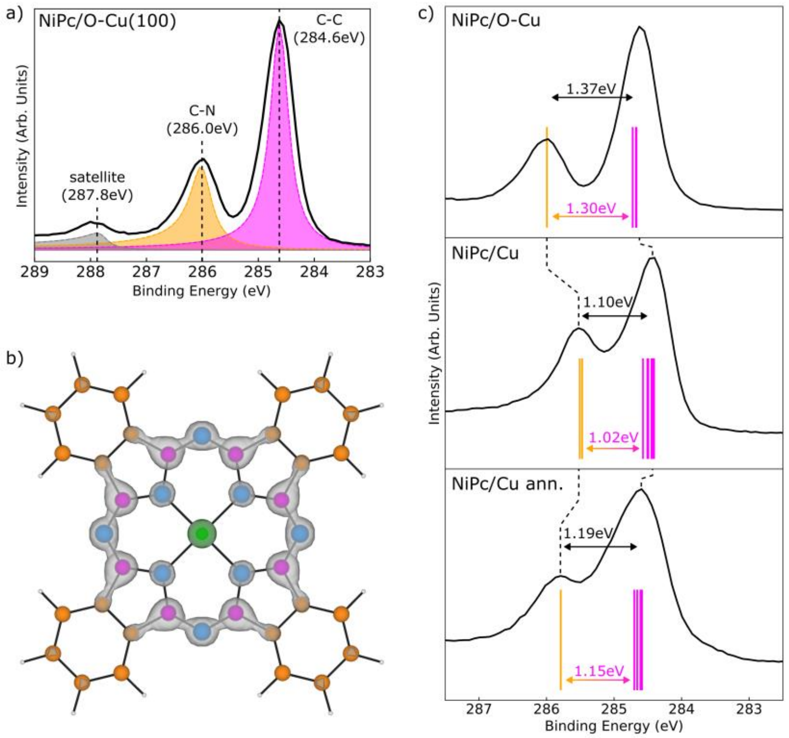

Figure S14. a) C1s XPS of NiPc/O-Cu(100) with experimental peak fits. b) Calculated electron density summed over LUMO and LUMO+1. c) Experimental C1s spectra of NiPc on passivated Cu and deposited on bare Cu before and after annealing; lines mark the calculated energies using the ΔK-S approach; absolute values are shifted for comparison with experiment.

S15. Ni $2p_{3/2}$ XPS spectra

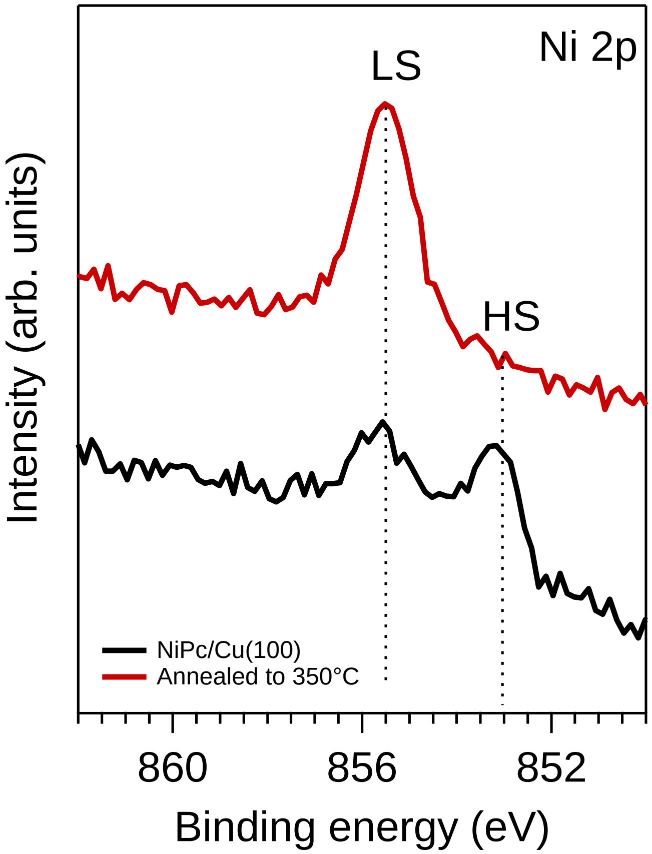

Figure S15. Ni $2p_{3/2}$ core-level XPS spectra acquired for NiPc/Cu(100) before and after annealing to 575 K, hv=1030 eV, p-pol.

	**HS**	**LS**
	**Coverage 0.5 ML**	
**Binding energy /eV**	853.22	855.59
**FWHM /eV**	1.67	1.56
**Area /arb.u.**	15.06	9.42
	**Coverage 0.9 ML**	
**Binding energy /eV**	853.16	855.51
**FWHM /eV**	1.45	1.86
**Area /arb.u.**	19.78	18.22
	**Coverage 1.1 ML**	
**Binding energy /eV**	853.18	855.54
**FWHM /eV**	1.42	1.81
**Area /arb.u.**	20.64	26.55

Table S2. Fitting parameters of the Ni $2p_{3/2}$ core-level XPS spectra acquired for NiPc/Cu(100) reported in Figure 2c.

References

[1] J. C. Buchholz, G. A. Somorjai, *J. Chem. Phys.* **1977**, *66*, 573–580.

[2] S. H. Chang, S. Kuck, J. Brede, L. Lichtenstein, G. Hoffmann, R. Wiesendanger, *Phys. Rev. B - Condens. Matter Mater. Phys.* **2008**, *78*.

[3] Q. Guo, Z. Qin, K. Zang, C. Liu, Y. Yu, G. Cao, *Langmuir* **2010**, *26*, 11804–11808.

[4] P. H. Lippel, R. J. Wilson, M. D. Miller, C. Wöll, S. Chiang, *Phys. Rev. Lett.* **1989**, *62*, 171–174.

[5] H. Okuyama, S. Kuwayama, Y. Nakazawa, S. Hatta, T. Aruga, *Surf. Sci.* **2022**, *723*.

[6] F. Chen, X. Chen, L. Liu, X. Song, S. Liu, J. Liu, H. Ouyang, Y. Cai, X. Liu, H. Pan, J. Zhu, L. Wang, *Appl. Phys. Lett.* **2012**, *100*.

[7] M. S. Liao, S. Scheiner, *J. Chem. Phys.* **2001**, *114*, 9780–9791.

[8] I. Cojocariu, H. M. Sturmeit, G. Zamborlini, A. Cossaro, A. Verdini, L. Floreano, E. D'Incecco, M. Stredansky, E. Vesselli, M. Jugovac, M. Cinchetti, V. Feyer, C. M. Schneider, *Appl. Surf. Sci.* **2020**, *504*, 144343.

[9] L. Ottaviano, S. Di Nardo, L. Lozzi, M. Passacantando, P. Picozzi, S. Santucci, *Surf. Sci.* **1997**, *373*, 318–332.

[10] F. Petraki, V. Papaefthimiou, S. Kennou, *Org. Electron.* **2007**, *8*, 522–528.

[11] A. Casotto, G. Drera, D. Perilli, S. Freddi, S. Pagliara, M. Zanotti, L. Schio, A. Verdini, L. Floreano, C. Di Valentin, L. Sangaletti, *Nanoscale* **2022**, *14*, 13166–13177.
